# Stimuli-Responsive Poly(aspartamide) Derivatives and Their Applications as Drug Carriers

**DOI:** 10.3390/ijms22168817

**Published:** 2021-08-16

**Authors:** Guangyan Zhang, Hui Yi, Chenhui Bao

**Affiliations:** 1 School of Materials and Chemical Engineering, Hubei University of Technology, Wuhan 430068, China; yihuihbut@163.com (H.Y.); xiaochengzikid@163.com (C.B.); 2 Hubei Provincial Key Laboratory of Green Materials for Light Industry, Hubei University of Technology, Wuhan 430068, China

**Keywords:** poly(aspartamide) derivatives, stimuli-responsive, drug carrier, nanoparticles, hydrogel, polymer-drug conjugate, drug-loading, controlled drug release

## Abstract

Poly(aspartamide) derivatives, one kind of amino acid-based polymers with excellent biocompatibility and biodegradability, meet the key requirements for application in various areas of biomedicine. Poly(aspartamide) derivatives with stimuli-responsiveness can usually respond to external stimuli to change their chemical or physical properties. Using external stimuli such as temperature and pH as switches, these smart poly(aspartamide) derivatives can be used for convenient drug loading and controlled release. Here, we review the synthesis strategies for preparing these stimuli-responsive poly(aspartamide) derivatives and the latest developments in their applications as drug carriers.

## 1. Introduction

Though the carbon-carbon backbone is conducive to the stability of polymers, it also limits the applications of these polymers in biomedical fields due to its low biocompatibility and non-biodegradability. In the past decades, the interest in amino acid-based biodegradable polymers has increased significantly in biomedicine due to their good biocompatibility, biodegradability and non-toxicity of their degradation products. For example, poly(glutamic acid) (PGA) and PGA-based polymers have been widely applied for controlled release of peptide, protein and anti-tumor drugs [1]; polylysine (PLL) and its derivatives have been extensively investigated in gene delivery systems [2]. Moreover, other amino-acid containing degradable polymers have also been studied in tissue engineering and biomedical imaging (e.g., magnetic resonance imaging and fluorescence imaging).

**Figure 1:**
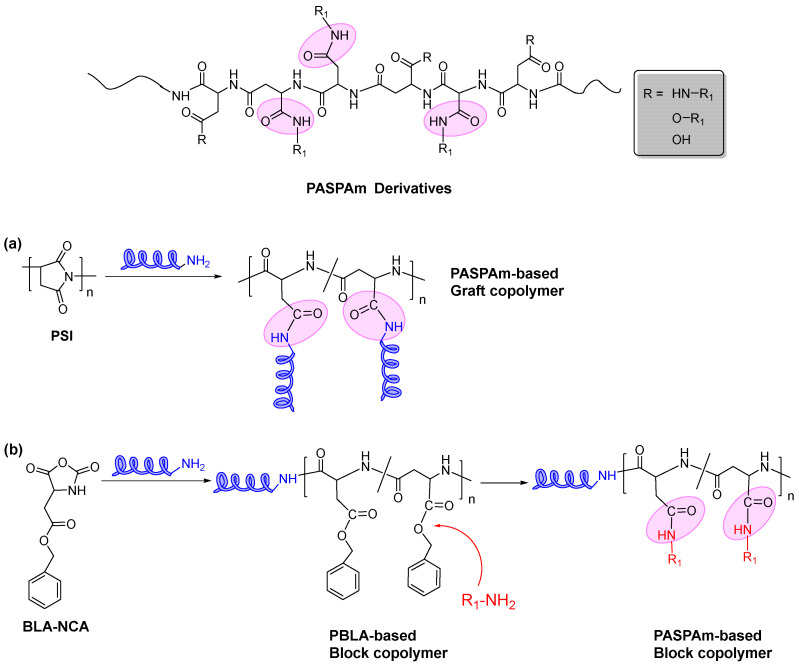
The synthetic pathways of PASPAm-based (**a**) graft copolymers and (**b**) block copolymers.

Poly(aspartic acid)-based polymers, whose backbone is composed of aspartic acid, are one of the most intensively studied amino acid-based polymers in drug [3,4]/gene [5,6,7] delivery systems and other biomedical applications [8] due to their simple synthesis method, excellent biocompatibility and biodegradability. The synthetic strategies of poly(aspartic acid)-based polymers mainly include the aminolysis reaction of poly(succinimide) (PSI) (Figure 1a) and the ring-opening polymerization (ROP) of benzyl-l-aspartate *N*-carboxyanhydride (BLA-NCA) (Figure 1b). For side chain-modified poly(aspartic acid)-based polymers, they can generally be prepared by the aminolysis reaction of PSI. For example, a side chain-modified poly(aspartamide) (PASPAm) derivative, α,β-poly(N-2-hydroxyethyl)-d,l-aspartamide (PHEA), can be obtained by the aminolysis reaction between ethanolamine and PSI, and has been further modified and intensively studied as scaffolds [9], siRNA delivery systems [10,11] and drug carriers (e.g., hydrogel, nanoparticles) [12,13,14,15,16]. For the block copolymers containing poly(aspartic acid)-based segment, they usually can be synthesized by ROP of BLA-NCA. For instance, NK105 polymer, which is composed of a poly(ethylene glycol) (PEG) block as the hydrophilic segment and modified poly(aspartic acid)-based block as the hydrophobic segment in which 50% carboxylic groups of PASPAm were reacted with 4-phenyl-1-butanol by esterification reaction [17,18], has been used as delivery system in a paclitaxel-incorporating micellar nanoparticle anti-cancer formulation (NK105) that was approved for clinical trials (phase III) [19,20]. Besides NK105, poly(aspartic acid)-based polymers were also applied in two other formulations, NK911 [21] and NC-6300 [22,23]. Among the above three clinical formulations, NC-6300 that is an epirubicin (EPI) conjugated PEG-block-PASPAm derived copolymer containing acid-labile hydrazone linkages, is the only one with stimuli-responsiveness. Results showed that NC-6300 exhibited a stronger anti-tumor effect and lower cardiotoxicity compared to free EPI, which may be due to its pH responsiveness.

Because stimuli-responsive polymers can respond to the changes of environmental factors (e.g., pH, temperature) by self-changing either their physical properties, chemical structures, or both, they exhibit attractive prospects in a variety of fields such as sensors, energy conversion and drug/gene delivery systems [24]. Therefore, various stimuli-responsive PASPAm derivatives such as NC-6300 have been designed and synthesized over the last decades, and their potential applications in different fields were also investigated extensively. In this review, PASPAm derivatives are defined as polymers composed wholly or partly of aspartamide units as shown in Figure 1.

Although some papers have reviewed the synthesis and applications of poly(aspartic acid) (PASP) [25] and PSI [26] derivatives, there is no comprehensive review summarizing the recent progress of “stimuli-responsive PASPAm derivatives”. In this paper, we will provide an overview of the up-to-date developments on stimuli-responsive PASPAm derivatives and their applications as drug carriers. First, the synthesis strategies of stimuli-responsive PASPAm derivatives will be reviewed by the type of triggers. Next, the applications of stimuli-responsive PASPAm derivatives for drug delivery will be introduced. Finally, the further perspectives of stimuli-responsive PASPAm derivatives will be discussed.

## 2. Synthesis

### 2.1. Temperature-Responsive Poly(aspartamide) Derivatives

Temperature is an important stimulus for stimuli-responsive polymers. Poly(*N*-isopropylacrylamide) (PNIPAAm) is one of the most investigated temperature-responsive polymers with a lower critical solution temperature (LCST) at around 32 °C. As is well known, the temperature-responsive behavior of PNIPAAm is ascribed to its *N*-isopropylamide pendants. Therefore, *N*-isopropylamide and various functional groups with similar structures were introduced for preparing temperature-responsive PASPAm derivatives. For instance, two different temperature-responsive PASPAm derivatives, PAIPAHA and PAA-TS, were prepared by the following procedures: (1) First, a certain amount of isopropylamine was reacted with PSI, and then (2) alkanolamines [27] or NaOH [28] were further used to open the remaining succinimide rings to obtain temperature responsiveness. Besides *N*-isopropylamide pendants, diisopropylamide [29] and isopropylethylenediamide [30,31] pendants were also introduced for the design of temperature-responsive PASPAm derivatives.

In fact, in the absence of isopropylamide or diisopropylamide pendants, PASPAm derivatives containing alkanolamides moieties also can possess temperature responsiveness. In 2003, a series of temperature-responsive PASPAm(C_5_OH/C_6_OH) were synthesized via the aminolysis reaction of PSI with a mixture of 5-aminopentanol (NH_2_C_5_OH) and 6-aminohexanol (NH_2_C_6_OH) by Kobayashi’s group [32]. Interestingly, the reaction product of PSI with NH_2_C_5_OH, denoted as PHPA, was completely soluble in water; while the reaction product of PSI with NH_2_C_6_OH was insoluble in water (test temperature: 0–100 °C). Since then, more and more temperature-responsive PASPAm derivatives containing alkanolamides moieties were designed and synthesized [33,34,35]. For instance, adopting the similar strategy of Kobayashi’s group, Chu et al. prepared a new family of temperature-responsive PASPAm(C_4_OH/C_6_OH) with an LCST ranging from 28 to 53 °C using the mixture of 4-aminobutanol (NH_2_C_4_OH)/NH_2_C_6_OH instead of the mixture of NH_2_C_5_OH/NH_2_C_6_OH [36]. In 2014, phenethyl alcohol (phe) was grafted to the pentanolamide pendants of PHPA, and the obtained five phe-g-PHPA polymers all exhibited temperature responsiveness. The LCST of phe-g-PHPA can be tuned by adjusting the percentage of grafted phe moieties, and has a negative linear correlation with the grafting ratio of phe [37]. In 2015, a certain amount of 2-azidoethylamine (Az) was used for aminolysis reaction with PSI, and then excess NH_2_C_5_OH was further utilized to open the residual succinimide rings to prepare azide-functional PASPAm derivative P(Asp-Az)x-HPA. Two P(Asp-Az)x-HPA polymers showed temperature-responsive behaviors in aqueous solution, and the molar ratio of Az/NH_2_C_5_OH in these two P(Asp-Az)x-HPA polymers was 39/61 and 56/44, respectively [38]. Additionally, the LCST of P(Asp-Az)x-HPA-based polymers can be further adjusted by grafting hydrophobic moieties, such as cinnamoyl group [33].

Moreover, ether oxygen also plays an important role for the design of temperature-responsive polymers. For example, oligo(ethylene glycol) (OEG) containing ether oxygen was usually introduced for preparing temperature-responsive polymers, such as OEG methacrylate based polymers [39,40] and polypeptides bearing OEG pendants [41,42]. In 2021, linear alkyl ether-type amine compounds, which contain one ether oxygen and varying carbon atoms, were successfully used for the synthesis of temperature-responsive PASPAm derivatives bearing alkyl ether-type pendants [43]. The composition of temperature-responsive PASPAm derivatives and their LCSTs are summarized in Table 1.

### 2.2. pH-Responsive Poly(aspartamide) Derivatives

Poly(aspartic acid) is a water soluble polymer, and its conformation in aqueous solution can be affected by pH due to pH-induced deprotonation and protonation of its carboxylic acid pendants. For example, the α helical conformation of poly(aspartic acid) that appeared in acidic solution was not observed in basic solution [44], which may be attributed to the negative charge repulsion caused by the deprotonation of pendant carboxylic acid groups in alkaline environment. Thus, one strategy for preparing pH-responsive PASPAm derivatives is to introduce other pH-sensitive groups or non-ionic groups. The introduction of non-ionic groups can usually be used to adjust the phase transition pH value (pH_t_) [45].

1-(3-aminopropyl) imidazole (API) is one of the most commonly used reagents for introducing imidazole groups to PASP or PBLA. For instance, Kim et al. grafted imidazole to PEG-b-PASP copolymer in which 60% carboxyl groups were reacted with the amine group of API forming amide linkages, resulting a pH-responsive zwitterionic PASPAm derivatives [46]. In addition, pH-responsiveness also can be obtained by substituting the benzyl groups of PBLA with API [47]. Another way for introducing imidazole groups to synthesize pH-responsive PASPAm derivatives is to directly react the succinimide rings of PSI with API [48,49,50,51,52,53] or histamine [54] through aminolysis reaction. Besides imidazole, tertiary amine [34,55,56,57,58] and other groups [30,59] are also intensively adopted for endowing pH responsiveness to PASPAm derivatives.

Another strategy for preparing pH-responsive polymers is to introduce pH-cleavable linkages, which mainly include hydrazone [60,61,62], imine [63], acetal, orthoester and 2,3-dialkylmaleamidic amide linkages. Hydrazone linkage [64] plays an important role in the preparation of pH-responsive polymers. The most well-known pH-responsive PASPAm derivative is NC-6300, in which EPI is bound to a PEG-PASPAm block copolymer through hydrazone linkage [23]. Thus, EPI can be released under acidic environments, such as in lysosomes. Doxorubicin (DOX)-conjugated PASPAm derivatives with hydrazone linkages were also designed and prepared by researchers [65,66,67,68]. Moreover, two PASPAm cross-linkers containing hydrazide groups (PHHZA) or amine groups (PHEDA) were synthesized by Cha’s team. PHHZA and PHEDA can react with the aldehyde groups of oxidized alginate forming alginate-based hydrogels via hydrazone and imine linkages, respectively [69]. In addition, Kataoka’s team synthesized three pH-responsive charge-conversional PASPAm derivatives containing cis-aconitic amide linkage (PAsp(DET-Aco) [70], PEG-PAsp(DET-Aco) [71]) or 2-propionic-3-methyl maleic amide linkage (PEG-PAsp(DET-PMM) [71]). The obtained three polymers showed negative charge at pH 7.4 due to the carboxylic group in cis-aconityl (Aco) or 2-propionic-3-methyl maleic (PMM) moieties, but exhibited positive charge at pH 5.5 because they can return to PAsp(DET) or PEG-PAsp(DET) via the breakup of cis-aconitic amide linkage or 2-propionic-3-methyl maleic amide linkage under acidic conditions. The pH-cleavable linkages used for preparing pH responsive PASPAm derivatives are summarized in Table 2.

Besides the two above mentioned strategies, the unreacted succinimide rings in PASPAm derivatives also can be utilized to design pH-responsive controlled-release delivery vehicles, because the hydrophobic succinimide units can be hydrolyzed at pH above 7.0 to form hydrophilic aspartic acid units, thereby releasing the encapsulated materials due to the hydrophobic-to-hydrophilic change of PASPAm derivatives [73].

Interestingly, it has been reported that some temperature-responsive PASPAm derivatives also exhibit pH responsiveness even without introducing pH-responsive functional groups or pH-cleavable linkages. The pH responsiveness of temperature-responsive PASPAm derivatives may be related to the presence of hydroxyl groups in alkanolamide pendants [36] or the end groups (carboxyl and amine groups) of PASPAm backbone [43].

### 2.3. Redox-Responsive Poly(aspartamide) Derivatives

**Figure 2:**
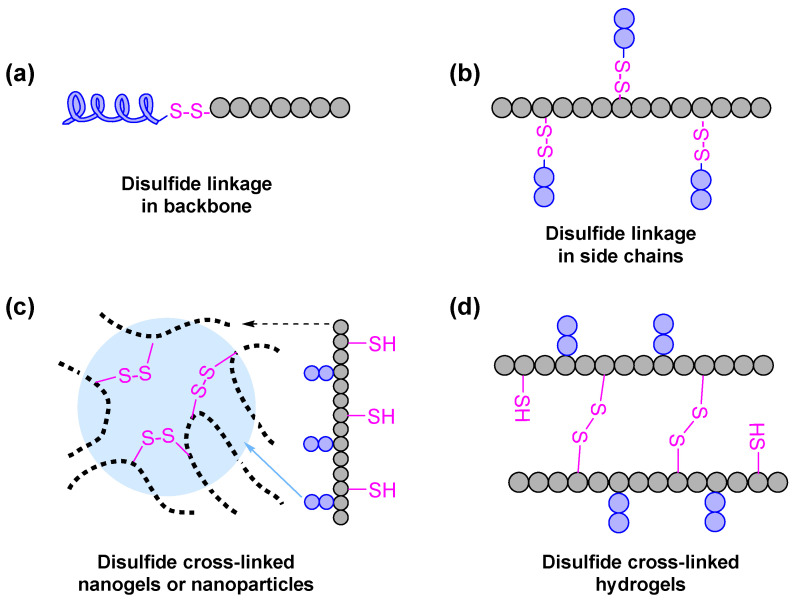
The redox-responsive PASPAm derivatives containing disulfide linkages (**a**) in backbone and (**b**) in side chains, and disulfide cross-linked (**c**) nanogels or nanoparticles and (**d**) hydrogels.

PASPAm derivatives generally can be endowed with redox-responsiveness by introducing disulfide linkage in their backbone (Figure 2a) or side chains (Figure 2b). Chu et al. [47] used cystamine-modified mPEG as a macroinitiator to initiate the polymerization of BLA-NCA, and then obtained a redox-responsive mPEG-block-PASPAm derivative, which contains a disulfide linkage between mPEG segment and PASPAm segment. Redox-responsive PASPAm derivatives, which have mPEG pendants with disulfide linkages, can also be synthesized through grafting 3,3’-dithiodipropionic acid-modified mPEG [74] or the aminolysis reaction between PSI and amino-terminated disulfide functionalized mPEG [48]. Moreover, some cationic polymers [75,76] or other compounds [77] can also be functionalized with cystamine and then used to design reduction-responsive PASPAm derivatives for DNA/RNA delivery. Of course, cystamine also can be used as a cross-linker for preparing reduction-responsive nanogels [78] or nanoparticles [33] (Figure 2c). In addition to S-S linkage, Se-Se linkage is also a good strategy for designing redox-responsive PASPAm derivatives [79].

In addition, thiol-disulfide inter-conversion can also be used for preparing redox-responsive PASPAm hydrogels [80,81,82,83] or stabilized disulfide cross-linked nanoparticles [84]. For instance, PSI first reacted with cysteamine, and then further reacted with other amine compounds (e.g., *N*,*N*-dimethyl-2-aminoethyl [85], 6-monodeoxy-6-monoamino-beta-cyclodextrin hydrochloride [86]) to obtain thiolated PASPAm derivatives. The obtained thiolated PASPAm derivatives can be cross-linked by the oxidation of the thiol groups in their cysteamide pendants and formed reduction-responsive hydrogels (Figure 2d).

### 2.4. Other Stimuli-Responsive Poly(aspartamide) Derivatives

Besides the above three stimuli-responsive (temperature, pH and redox) PASPAm derivatives, PASPAm derivatives with other stimuli responsiveness (including light responsiveness and carbon dioxide (CO_2_) responsiveness) have also been investigated in the past two decades.

CO_2_ is an inexpensive gas and also an important metabolite in cells. It is reported that polymers containing dialkylamine [87,88], amidine [89] or guanidine [90] moieties can exhibit reversible CO_2_-responsive behavior because CO_2_ can be bonded onto these polymers in the presence of water. For example, Kim’s group prepared three CO_2_-responsive PASPAm derivatives, PHEA-HIS [54], PHEA-Larg and PolyAspAm(OA/Larg) [91]. The swelling degree of cross-linked PHEA-HIS hydrogel decreased to about 18 when CO_2_ was bubbled into the solution containing PHEA-HIS hydrogel samples, and returned to about 50 after bubbling N_2_ gas. PHEA-Larg hydrogels and PolyAspAm(OA/Larg) nanoparticle also exhibited similar reversible volume change behavior by CO_2_/N_2_ purge.

Because light is a clean stimulus and can be easily triggered on or off, light-responsive polymers have shown huge potential for a variety of applications. For designing light-responsive polymers, photo-cleavage groups such as o-nitrobenzyl [92] were generally introduced. For instance, o-nitrobenzyl (NB) alcohol [35] was first activated by *N*,*N′*-carbonyldiimidazole and then grafted onto the pendant pentanolamide moieties of PHPA. When the obtained light-responsive NB-g-PHPA was treated under UV light at 365 nm for 30 min, the photo-cleavage o-nitrobenzyl carbonate ester linkage was broken and byproduct o-nitrosobenzaldehyde was detected.

In addition, light can also be used for cross-linking to stabilize PASPAm-based micelles. For instance, light responsive coumarin moieties were introduced to the side chains of PASPAm derivative, and the formed micelles can be conveniently cross-linked and stabilized by exposing it to the light of 365 nm for 30 min [93]. Photo-crosslinking can also be achieved by introducing methacryloyl [9,94] or acryloyl [95] moieties to the ethanolamide or hydrazide pendants of PASPAm.

Dual or multi-responsive PASPAm derivatives can also be prepared by the combination of above-mentioned strategies for endowing various stimuli-responsiveness. Therefore, we will not go into the details here.

## 3. Applications

Due to their good biocompatibility and biodegradability, PASPAm derivatives could be regarded as potential and promising materials and can be utilized in various areas of biomedical applications, such as drug carriers. As a drug carrier, the process of drug loading and drug release are both important. PASPAm derivatives with stimuli-responsiveness can usually show convenient drug loading methods or excellent controlled drug release performances. Therefore, this part focuses on the applications of stimuli-responsive PASPAm derivatives as drug carriers from two aspects: drug loading and drug release.

### 3.1. Drug Loading

Amphiphilic polymers, which can self-assemble to form nanoparticles including micelles and vesicles, are one of the most important classes of polymers used to deliver hydrophobic drugs such as paclitaxel (PTX) and doxorubicin (DOX). For preparing drug-loaded nanoparticles, one of the important methods is dialysis. However, the dialysis method often takes a long time (at least a few hours) and requires the use of toxic organic solvents (e.g., DMSO, CH_3_OH) to dissolve the polymeric carrier [93], and the drug loading efficiency is usually low. Thus, a simple, easy-to-implement drug loading strategy with high efficiency is needed for preparing drug-loaded nanoparticles.

**Figure 3:**
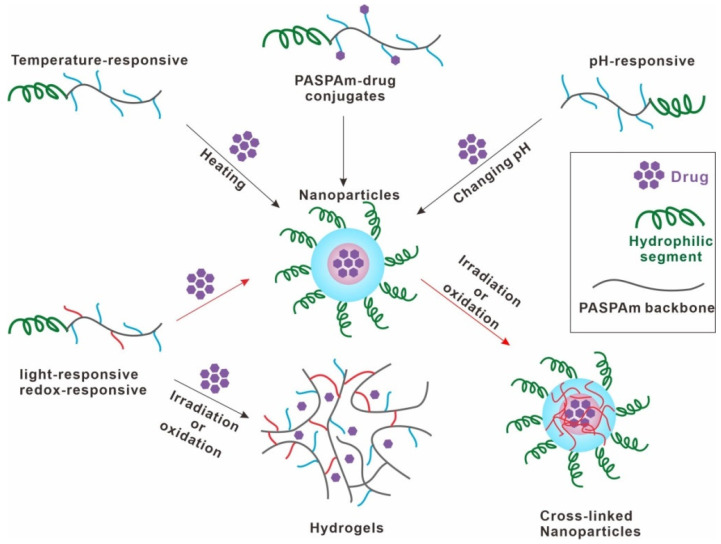
Typical drug loading procedures of various stimuli-responsive PASPAm derivatives.

Temperature and pH are often used as triggers for drug loading. For temperature responsive polymers, the general drug loading process is as follows: (1) prepare polymer aqueous solution of a certain concentration in PBS at a temperature (e.g., 4 °C) lower than LCST; (2) add high-concentration drug solution (e.g., 20 mg/mL) to the prepared temperature responsive polymer aqueous solution; (3) heat the mixture of drug solution and polymer aqueous solution immediately with about 1 min incubation by soaking into a water bath or other ways (temperature higher than LCST, such as 60 °C); (4) slowly cool down the mixture to room temperature and then filter the mixture to remove non-entrapped hydrophobic drug. The process of preparing drug-loaded nanoparticles from the temperature-responsive polymer aqueous solution is called the quick heating method. For example, Jiang et al. reported that PTX was successfully loaded into micelles formed by temperature-responsive PASPAm derivatives through quick heating method, avoiding the use of toxic organic solvents [35,96,97]. Using temperature-responsive PEGylation phe-g-PHPA as drug carrier, the PTX-loading capacity was high, up to 29%, with a high loading efficiency of 99% [97]. Using pH-responsive PASPAm derivatives, the drug also can be loaded into micelles by quickly changing the pH of the polymer aqueous solution from below to above pH_t_. For instance, the aqueous mixture containing pH-responsive phe/DEAE-g-PHPA-g-mPEG and doxorubicin hydrochloride was prepared at pH value below pH_t_ first (pH = 4.0), and then a certain volume of weakly alkaline PBS solution (pH = 8.2) was added to adjust the pH above pH_t_. After filtering to remove non-entrapped DOX, DOX-loaded micelles prepared with pH-responsive phe/DEAE-g-PHPA-g-mPEG were obtained [34]. Therefore, temperature-responsive and pH-responsive PASPAm derivatives play an important role in the drug loading process because they can achieve a rapid drug loading procedure (Figure 3).

Hydrogels are also an important class of polymers that can be used as drug carriers. Cross-linking is a well-known strategy for preparing drug-loaded hydrogels or nanogels. The cross-linking reaction can occur between two polymers containing reactive functional groups [13,69,81], or between polymer and small molecule cross-linker [98,99,100,101]. Of course, enzymes can also be used as cross-linker to promote the cross-linking reaction of PASPAm derivatives for the preparation of nanogels [102]. For PASPAm derivatives without stimuli-responsiveness, another option is the precipitation of the polymer in physiological medium to form gel-like depot. The drug and polymer can be dissolved in an organic solvent with high miscibility with water, and then the resulting mixture solution was slowly injected into physiological medium to allow the formation of drug-loaded hydrogels. According to this strategy, Fiorica et al. successfully prepared a sulpiride-loaded gel-like depot using PASPAm-polylactide copolymer, in which *N*-methyl-2-pyrrolidone was employed to dissolve sulpiride (an antipsychotic drug) and PASPAm-polylactide copolymer due to its low systemic toxicity [14]. This sulpiride-loaded depot can prolong a sustained release of sulpiride in vitro for about one week.

For PASPAm derivatives that respond to stimuli, even if organic solvents are not used, drug-loaded hydrogels can also be prepared, which will reduce the potential risks caused by the use of organic solvents. For instance, Cao et al. successfully prepared ketoprofen-loaded hydrogels by irradiation of the mixture of light-responsive PASP-Hy-AC aqueous solution and ketoprofen (a nonsteroidal anti-inflammatory drug) under long-wavelength UV for 1 h, and the obtained ketoprofen-loaded hydrogel exhibited the sustained ketoprofen release for about 50 h [95]. Redox-responsive PASPAm derivatives containing thiol groups can also be used for drug-loading. For example, PASP-CEA was dissolved in the PBS containing ofloxacin (a second-generation fluoroquinolone antibiotic), and then oxidant solution was added to ofloxacin/PASP-CEA PBS solution to form ofloxacin-loaded hydrogel via the oxidation of the thiol groups [83]. Moreover, some temperature-responsive polymer aqueous solutions can turn into a gel state in a short time (a few minutes) when heated. Thus, the formation of drug-loaded hydrogels based on temperature-responsive polymers, such as chitosan/*β*-glycerophosphate [103,104] and Pluronic F127 [105], can be triggered by the temperature difference between the external environment and the body. However, there is no report on the use of temperature-responsive PASPAm derivatives for preparing drug loaded hydrogels, although there are many reported temperature-responsive PASPAm derivatives.

Polymer-drug conjugates composed of drug molecules covalently linked to polymeric carriers are also an important part of drug delivery systems. Because the drug and the polymer carrier are covalently linked, polymer-drug conjugates generally do not require response to external stimuli to achieve drug loading.

### 3.2. Drug Release

As drug carriers, it is also very important that they can release drugs to the desired locations in response to specific stimuli. Thus, stimuli-responsive polymers also play a key role in the drug release process. For drug release, pH and redox are the most investigated triggers due to the acidic environment around the tumor (pH range of 5.5–7.0) [106] and in the endosome (pH ≈ 5.0) [107] and the presence of reduced glutathione (GSH) [108].

Although polymer-drug conjugates are usually not required to respond to external stimuli during drug loading, they are often required to respond to external stimuli during the drug release process to deliver the drug to the desired location. NC-6300, which has already undergone Phase I (19 subjects) [109] and Phase 1b (29 subjects) [110] clinical trials, is the most famous stimuli-responsive polymer-drug conjugate based on PASPAm. The EPI conjugated with hydrazone linkage in NC-6300 can be released under acidic environment around tumors, so it exhibits selective tumor accumulation. Clinical trial results showed that NC-6300 was well tolerated in patients with various solid tumors including urothelial carcinoma, breast cancer, cholangiocarcinoma and leiomyosarcom, and its toxicity was lower than conventional EPI formulations. The maximum tolerated dose and recommended phase 2 dose of NC-6300 obtained in Phase 1b were 185 mg/m^2^ and 150 mg/m^2^, respectively, which are higher than conventional EPI dose. Besides EPI, DOX was another widely investigated anti-tumor drug in polymer-drug conjugates as a drug delivery system. For instance, Lim et al. conjugated DOX to alendronate (ALN)-modified PASPAm derivatives with hydrazone linkages to obtain polymer-DOX conjugate ALN-PEG/C18/HYD-DOX-g-PASPAM, and prepared bone targeting nanoparticles. Results showed that 75% of the conjugated DOX was released from the prepared polymer-DOX conjugate at pH 5.0 due to the cleavage of hydrazone bonds, and the volume of tumor decreased significantly to 1550 mm^3^ after treatment with ALN-PEG/C18/HYD-DOX-g-PASPAM (control sample in PBS, 3850 mm^3^) [66]. The pH-controlled release of DOX in other PASPAm-DOX conjugates containing hydrazone linkages were also studied, results showed that the amount of DOX released at pH 5.0 was significantly more than that at pH 7.4 in the same time period [65,67,68]. Chang et al. conjugated PTX to the end group (-NH_2_) of the backbone of PEG-b-PBLA via a reduction-responsive disulfide linkage using 3,3′-dithiodipropionic acid as a coupling agent, and results showed that the release rate of PTX in an environment with 10 mM GSH (mimicking the environment in tumor cells) was significantly higher than that in an environment with 2µM GSH (mimicking the environment in human blood) [111]. Therefore, the introduction of stimuli-responsive cleavable linkages is a key strategy for the controlled drug release of polymer-drug conjugates.

For nanoparticle drug delivery systems prepared from amphiphilic polymers, the response to environmental stimuli generally can break the hydrophobicity-hydrophilicity balance, thereby disturbing the stability of the drug-loaded nanoparticles to achieve the aim of drug release. Gu et al. prepared DOX-loaded nanoparticles by dialysis using temperature-responsive PAIPAHA as polymeric carrier, and their DOX release profiles evaluated at different temperature showed that the release amount and rate of DOX at 37 °C (human body temperature, 45% within 10 h) were both higher than that at 25 °C (room temperature, 35% after 110 h) [27]. This may be caused by the LCST of PAIPAHA (30 °C) being between room temperature and body temperature. When the environmental temperature is higher than the LCST of PAIPAHA, the formed nanoparticles were disturbed and became unstable due to the weakened hydrogen-bonding or hydrophobic interactions between PAIPAHA and DOX. Thus, temperature can be used as a switch to control the drug release. The response to pH or GSH can also be used to realize the controlled release of drug from nanoparticles. Cai et al. prepared a disulfide cross-linked micellar nanodrug loaded with sorafenib from pH/reduction dual-responsive mPEG-PAsp (MEA&DIP), and realized controlled release of sorafenib using pH and GSH as the switch [112]. Due to the disulfide cross-linking, this sorafenib-loaded nanodrug can remain stable in blood circulation, but it can also rapidly release sorafenib inside cancer cells due to GSH-induced disulfide bond breakage and the protonation of DIP (2-aminoethyldiisopropylamide) pendants in an acidic environment (leading to a hydrophobic-to-hydrophilic change). Light with specific wavelength also can be used as a stimulus for controlled drug release. After 15 min of irradiation at 365 nm, PTX-loaded nanoparticles containing photo-cleavable o-nitrobenzyl carbonate ester linkages showed an significantly faster PTX release behavior [35].

For hydrogels, drug release generally can be controlled by the degree of swelling, and redox is one of the most investigated triggers due to the reductive environment in tumor tissue (10 mM GSH). Krisch et al. chose fluorescein isothiocyanate-dextran as model drug to investigate the drug release behavior. Results reveal that a reductive environment can promote the release of model drug from S-S cross-linked PASPAm hydrogel, because the degree of swelling increased (10 mM DTT in PBS) due to the cleavage of S-S linkages [81]. Park et al. prepared DOX-loaded PASPAm nanogels, the release rate of DOX in reductive environment was significantly faster than that in non-reductive environment due to the disintegration of nanogels [78].

## 4. Degradation

The degradability of PASPAm derivatives is based on the hydrolysis process of amide linkages in their backbone. Many studies have confirmed that the backbone of PASPAm derivatives can be degraded by bacteria (i.e., strain KP-2: JCM10638, 15 days) [113,114], enzyme (i.e., hydrolase purified from *Sphingomonas* sp. KT-1, *β*-amide linkage) [115,116] or activated sludge (28 days) [117,118] after a few days. For PASPAm derivatives that respond to stimuli, the degradation site and behavior are more diverse. For instance, Juriga et al. [119,120] investigated the degradability of cross-linked PASPAm hydrogels containing disulfide linkages with different enzymes (trypsin-EDTA, dispase and collagenase I). Collagenase I showed the best effect for the degradation of hydrogel disk, followed by trypsin-EDTA and then dispase, because collagenase I cleaved the disulfide linkages in the hydrogel. Interestingly, the degradation of PASPAm derivatives containing pH-responsive amino groups can be achieved without enzyme due to the nucleophilic attack of the N in CONH_side_ at the C in CONH_main_ [121], and can be affected by their pendant moieties. Naito et al. [7] reported that the bearing pendants with a longer alkyl spacer (i.e., 3-aminopropylamide (3 carbons), 4-aminobutylamide (4 carbons)) showed more strongly suppressed degradation than that with a shorter alky spacer (i.e., 2-aminoethylamide (2 carbons)). Although the degradation of PASPAm derivatives has been widely investigated, so far there have been no in vivo studies on their degradation.

## 5. Conclusions

With the ever-increasing demand for novel smart materials in biomedical fields, the development of stimuli-responsive PASPAm derivatives has attracted more and more attention due to their biocompatibility and biodegradability. From a synthesis point of view, most stimuli-responsive PASPAm derivatives can be easily prepared by introducing functional groups or moieties that respond to various stimuli, but it is worth noting that temperature-responsive PASPAm derivatives can also be synthesized without introducing specific temperature-responsive moieties. In addition, some temperature-responsive PASPAm derivatives possess pH responsiveness even without introducing pH-responsive functional groups or pH-cleavable linkages. Thus, the pH response mechanism of temperature-responsive PASPAm derivatives needs to be studied in depth in the future.

From an application point of view, stimuli-responsive PASPAm-based drug delivery systems including nanoparticles, hydrogels and polymer-drug conjugates are reviewed in this paper from two aspects: drug loading and controlled release. External stimuli can not only be utilized to achieve convenient drug loading but also controlled drug release. Temperature and pH generally can be used as triggers to realize drug loading for nanoparticles, while thiol groups and light with specific wavelength usually can be used as triggers to prepare drug-loaded hydrogels. Although temperature has been reported to trigger the preparation of drug-loaded hydrogels by other temperature-responsive materials, there is no report on the use of temperature-responsive PASPAm derivatives to prepare drug-loaded hydrogels. Considering the advantages of the stimuli-triggered drug loading method (avoiding the use of toxic organic solvents and high drug loading efficiency), such studies may be an interesting area in drug delivery systems. For controlled drug release, it can be triggered by most of stimuli, such as temperature, pH, GSH and light. NC-6300, which exhibits selective release of EPI into tumor tissue, is a typical representative of stimuli-responsive polymer-drug conjugates for drug delivery based on PASPAm derivatives.

In conclusion, stimuli-responsive PASPAm derivatives hold great prospects as drug carriers. Although some considerable progress has been made, there are still some issues that have not yet been clarified in terms of response mechanisms. Taking account of the growing interest in stimuli-responsive polymers, more interesting stimuli-responsive PASPAm derivatives may be designed and applied for drug delivery in the future.

## Figures and Tables

**Table 1:** A summary of temperature-responsive PASPAm derivatives.

Temperature Responsive PASPAm Derivatives	Composition of Side Chain		R_1_/R_2_	LCST (°C)	Ref
	R_1_	R_2_			
PAIPAHA	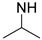	NH-(CH_2_)_6_OH	55/45	30	[27]
PAIPAPA	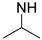	NH-(CH_2_)_3_OH	55/45	40	[27]
PAA-TS	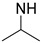	OH	69/31	38 ^a^	[28]
			73/27	40 ^a^	
			74/26	45 ^a^	
			75/25	47 ^a^	
			77/23	49 ^a^	
			78/22	56 ^a^	
			80/20	62 ^a^	
PSI-TS	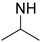	-	69/31	34 ^a^	[28]
mPEG-hyd-PDAHy	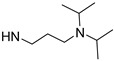	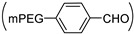 NH-NH_2_	80/(9)20	40	[29]
PolyAspAm(HA/NIPEDA)	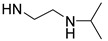	NH-(CH_2_)_5_CH_3_	57/43	24	[30]
			58/42	30	
			59/41	39	
			62/38	52	
PolyAspAm(OA/NIPEDA)	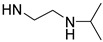	NH-(CH_2_)_7_CH_3_	57/43	22	[30]
			59/41	36	
			60.5/39.5	43	
			62/38	49	
PolyAspAm(LA/NIPEDA)	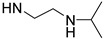	NH-(CH_2_)_11_CH_3_	59/41	22	[30,31]
			61/39	37	
			64/36	50	
PAspAm(C5OH/C6OH)	NH-(CH_2_)_5_OH	NH-(CH_2_)_6_OH	50/50	23	[32]
			60/40	29	
			70/30	37	
			80/20	44	
PAspAm(C4OH/C6OH)	NH-(CH_2_)_4_OH	NH-(CH_2_)_6_OH	30/70	53	[36]
			25/75	40	
			20/80	38	
			15/85	32	
			10/90	28	
phe-g-PHPA	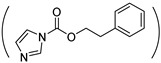	NH-(CH_2_)_5_OH	(2)/100	54	[37]
			(6)/100	32	
			(11)/100	20	
			(12)/100	14	
			(13)/100	9	
Phe/DEAE-g-PHPA	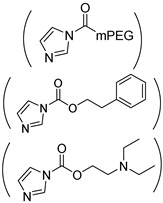	NH-(CH_2_)_5_OH	(0/22.2/30.3)/100	35.7 ^b^	[34]
			(0/26.6/28.3)/100	31.5 ^b^	
			(0/37.6/27.3)/100	22.5 ^b^	
			(0/39.6/19.0)/100	29.5	
			(0/53.6/12.0)/100	45.8	
			(5/34.6/12.7)/100	28 ^b^	
			(5/42.4/10.5)/100	15 ^b^	
NB-g-PHPA	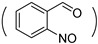	NH-(CH_2_)_5_OH	(4)/100	32	[35]
			(7.5)/100	21	
			(10)/100	11	
NB-g-PHPA-g-mPEG	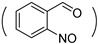 NH-(CH_2_)_5_OH	NH-mPEG	(12)98.5/1.5	25	[35]
			(17)98.5/1.5	8	
P(Asp-Az)x-HPA	NH-(CH_2_)_2_N_3_	NH-(CH_2_)_5_OH	40/60	63	[33]
			39/61	58	[38]
			56/44	29	
P(Asp-Az)x-HPA-PEA	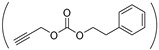 NH-(CH_2_)_2_N_3_	NH-(CH_2_)_5_OH	(5)39/61	19	[38]
			(9.9)39/61	4	
P(Asp-Az)x-HPA-IMZ	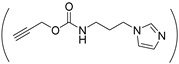 NH-(CH_2_)_2_N_3_	NH-(CH_2_)_5_OH	(5)39/61	22	[38]
			(9.7)39/61	10	
P(HPA-Az)-CA	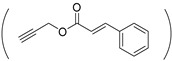 NH-(CH_2_)_2_N_3_	NH-(CH_2_)_5_OH	(2.5)40/60	47	[33]
			(4.3)40/60	40	
PASP-OCx	NH-(CH_2_)_3_OCH_2_CH_3_	-	100/-	54	[43]
	NH-(CH_2_)_2_OCH_2_CH_3_	-	100/-	87	

^a^ Determined at pH = 3.4; ^b^ Determined at pH = 7.4 PBS. mPEG: monomethoxy poly(ethyleneglycol).

**Table 2:** A summary of pH-responsive PASPAm derivatives containing pH-cleavable linkages.

pH Responsive PASPAm Derivatives	Linkages	Formation of Linkages between		Ref
		A	B	
NC-6300	Hydrazone	Hydrazide group of PEG polyaspartate block copolymer	Ketone group of EPI	[23]
Cross-linked nanocapsules with PADH and PACA	Hydrazone	Hydrazide group of PADH	Aldehyde groups of PACA	[56]
PALHy-hyd-DOX	Hydrazone	Hydrazide group of PALHy	Ketone group of DOX	[65]
ALN-PEG/C-18/HYD-DOX-g-PASPAM	Hydrazone	Hydrazide group of PASPAM	Ketone group of DOX	[66]
MPEG/CA10/DOX-g-PASPAM	Hydrazone	Hydrazide group of MPEG/Hyd/CA10-g-PASPAM	Ketone group of DOX	[67]
PA	Hydrazone	Hydrazide group of biotin-PEG/C18-PSI/Hydrazine	Ketone group of DOX	[68]
PEG/Hyd-Curcumin/C_18_-g-PSI (NFA-Cur)	Hydrazone	Hydrazide group of PEG/C18-g-PSI	Ketone group of Curcumin	[60]
Injectable PAsp hydrogel	Hydrazone	Hydrazide group of PAHy	Aldehyde groups of PAAld	[61]
PHHZA-linked alginate hydrogels	Hydrazone	Hydrazide group of PHHZA	Aldehyde groups of oxidized alginate	[69]
PHEDA-linked alginate hydrogels	Imine	Amino group of PHEDA	Aldehyde groups of oxidized alginate	[69]
Pasp-EDA-g-Ad/mPEG	Imine	Amino group of Pasp-EDA	Aldehyde group of 4-adamantane carboxylate benzaldehyde	[63]
PAsp(DET-Aco)	cis-Aconitic amide	Amino group of PAsp(DET)	Anhydride group of cis-aconitic anhydride	[70]
PEG-PAsp(DET-ACO)	cis-Aconitic amide	Amino group of PEG-PAsp(DET)	Anhydride group of cis-aconitic anhydride	[71]
PEG-PAsp(DET-PMM)	2-propionic-3-methyl maleic amide	Amino group of PEG-PAsp(DET)	Anhydride group of 2-propionic-3-methyl maleic anhydride	[71]
mPEG-g-P(ae-Asp)-Hap	β-carboxylic amide	Amino group of PEG-g-P(ae-Asp)	Anhydride group of hexahydrophthalic anhydride	[72]

## Data Availability

Not applicable.
